# Revealing the principles of inter- and intra-domain regulation in a signaling enzyme via scanning mutagenesis

**DOI:** 10.1101/2024.05.13.593907

**Published:** 2024-07-24

**Authors:** Ziyuan Jiang, Anne E. van Vlimmeren, Deepti Karandur, Alyssa Semmelman, Neel H. Shah

**Affiliations:** 1 Department of Chemistry, Columbia University, New York, NY 10027; 2 Department of Biological Sciences, Columbia University, New York, NY 10027; 3 Department of Biochemistry, Vanderbilt University, Nashville, TN 37232

**Keywords:** SHP2, *PTPN11*, tyrosine phosphatase, deep mutational scanning, allostery, molecular dynamics

## Abstract

Multi-domain enzymes can be regulated by both inter-domain interactions and structural features intrinsic to the catalytic domain. The tyrosine phosphatase SHP2 is a quintessential example of a multi-domain protein that is regulated by inter-domain interactions. This enzyme has a protein tyrosine phosphatase (PTP) domain and two phosphotyrosine-recognition domains (N-SH2 and C-SH2) that regulate phosphatase activity through autoinhibitory interactions. SHP2 is canonically activated by phosphoprotein binding to the SH2 domains, which causes large inter-domain rearrangements, but autoinhibition can also be disrupted by disease-associated mutations. Many details of the SHP2 activation mechanism are still unclear, the physiologically-relevant active conformations remain elusive, and hundreds of human variants of SHP2 have not been functionally characterized. Here, we perform deep mutational scanning on both full-length SHP2 and its isolated PTP domain to examine mutational effects on inter-domain regulation and catalytic activity. Our experiments provide a comprehensive map of SHP2 mutational sensitivity, both in the presence and absence of inter-domain regulation. Coupled with molecular dynamics simulations, our investigation reveals novel structural features that govern the stability of the autoinhibited and active states of SHP2. Our analysis also identifies key residues beyond the SHP2 active site that control PTP domain dynamics and intrinsic catalytic activity. This work expands our understanding of SHP2 regulation and provides new insights into SHP2 pathogenicity.

## Introduction

The majority of eukaryotic signaling proteins contain multiple domains with distinct biochemical functions. These domains facilitate catalytic activity, such as phosphorylation and dephosphorylation, or binding to ligands, including short linear protein motifs^1^, phosphorylated protein residues^2^, lipids^3^, and nucleic acids^4^. Eukaryotic proteins have evolved to harbor various combinations of these domains, resulting in a diverse array of fine-tuned multifunctional signaling modules^5,6^. A key feature of many modular, multi-domain signaling proteins is that they are highly regulatable switches capable of accessing different conformations in response to specific signals^7^. This is typically achieved through regulatory interactions between different domains and inter-domain linkers, which couple the functions of individual domains^8,9^. A classic example is the Src kinase module, which contains a polyproline-binding SH3 domain, phosphotyrosine-binding SH2 domain, and protein kinase domain^10^. The SH3 and SH2 domains allosterically suppress kinase activity through inter-domain interactions, and engagement of these domains by ligands simultaneously localizes the kinase and causes structural rearrangements that unleash its catalytic activity. The tight regulation of multi-domain signaling proteins also makes them highly prone to dysregulation by mutations, as the disruption of intra-domain regulation, inter-domain interactions, or linker structure and dynamics could readily shift the conformational balance toward an inactive or hyperactive state^11^. Thus, a complete understanding of multi-domain signaling protein regulation and dysregulation requires in-depth examination of each individual domain, as well as the allosteric network between domains in the whole protein.

**Figure 1.**
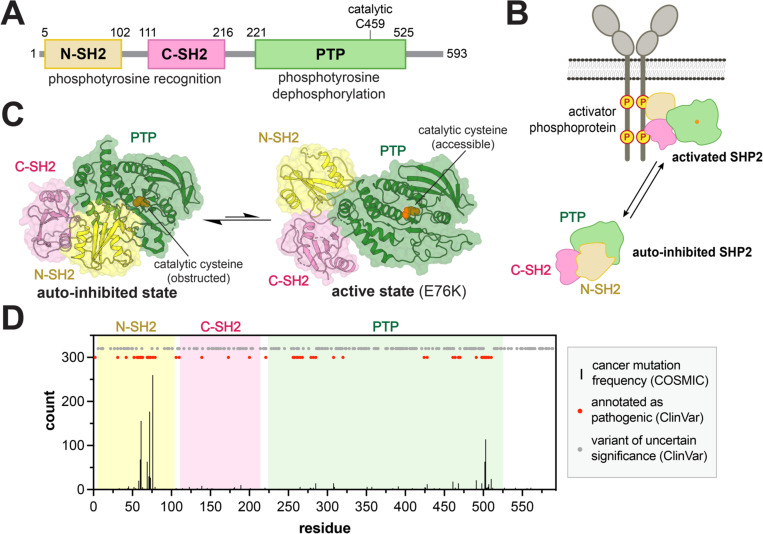
SHP2 activation and dysregulation by mutations. (**A**) Domain architecture diagram of SHP2. (**B**) Cartoon depiction of SHP2 activation by phosphoprotein binding. (**C**) Structures of the autoinhibited (PDB code 4DGP) and active states of SHP2 (PDB code 6CRF). Note that the active state shown is that of the E76K mutant, and other active conformations likely exist in solution. (**D**) Positions and frequencies of cancer hotspot mutations (black bars) in SHP2 along its 593 residue sequence. Sites of other pathogenic mutations and variants of uncertain significance are labeled as red and gray dots, respectively.

Protein tyrosine phosphatases represent an important class of multi-domain signaling proteins that have diverse domain architectures. They usually consist of a catalytic protein tyrosine phosphatase (PTP) domain alongside one or more binding domains, such as SH2, Sec14, PDZ, Bro1, and FERM domains^12^. SHP2 is a well-studied tyrosine phosphatase that harbors two SH2 domains before its PTP domain (Figure 1A). In signaling pathways such as the MAPK/Erk and Jak/Stat pathways, SHP2 is recruited via its SH2 domains to phosphotyrosine-bearing sequences on receptors and scaffold proteins (Figure 1B). Upon phosphoprotein binding, SHP2 switches from a closed, autoinhibited state characterized by extensive N-SH2/PTP domain interactions, to an open, active state, allowing for the dephosphorylation of downstream substrates (Figure 1B,C)^13^. SHP2 itself is also a target of tyrosine phosphorylation. Y62 on the N-terminal SH2 (N-SH2) domain and Y542 and Y580 on the disordered C-terminal tail are known phosphosites that likely regulate SHP2 by altering inter-domain interactions^14–16^. Extensive biochemical and biophysical studies on SHP2 have provided numerous insights into its structure and regulation. However, several details of the molecular mechanisms underlying SHP2 regulation remain unclear. Although there are many crystal structures of the autoinhibited wild-type SHP2 and one structure of an open conformation SHP2 mutant^17,18^, several studies indicate that activated SHP2 likely exists as an ensemble of open conformations, and the key structural features of this ensemble remain elusive^19,20^. Additionally, the transition pathway from the closed to open conformation(s) is not known. Furthermore, other tyrosine phosphatases have been shown to have intra-domain allostery within their PTP domain^21,22^. It is unclear whether this dynamic feature is intact in SHP2, nor whether that regulation is affected by SH2/PTP interactions.

SHP2 is prone to pathogenic mutations, which result in cancers and developmental disorders, such as juvenile myelomonocytic leukemia (JMML), Noonan syndrome, and Noonan Syndrome with multiple lentigenes^23^. Of the roughly 500 SHP2 mutants recorded in ClinVar and COSMIC, less than 200 are annotated as pathogenic, and they are distributed across all three domains of SHP2 (Figure 1D)^24,25^. Characterization of the molecular mechanisms underlying the pathogenicity of several of these mutants has provided insights into SHP2 regulation. For example, the well-studied E76K mutation at the N-SH2/PTP interface is highly activating, illustrating the importance of this autoinhibitory interface^18^. The T42A mutation in the N-SH2 ligand binding pocket alters SHP2 ligand affinity and specificity, sensitizing SHP2 to activators^26,27^. The Y279C mutation disrupts phosphoprotein binding in the PTP active site, thereby significantly diminishing catalytic activity^28^. However, not all pathogenic SHP2 mutations are well-characterized, and the molecular basis for their pathogenicity remains unclear. Furthermore, several hundred of mutations documented in ClinVar are characterized as variants of uncertain significance (Figure 1D)^24^. A comprehensive profiling of SHP2 mutational effects will shed light on the functional effects of these less studied SHP2 mutants, and in turn shed light on SHP2 allosteric regulation and dynamics.

Deep mutational scanning is a powerful method for characterizing protein mutants^29^. By combining selection assays on pooled libraries with deep sequencing, this method provides a way to profile mutational effects across a protein with high throughput. Deep mutational scanning has been applied to reveal structure-function relationships^30^, predict protein structures and dynamics^31,32^, map drug resistance^33^, and examine stability and expression^34,35^. Here, we present a deep mutational scanning platform to characterize the effects of SHP2 mutations on phosphatase activity. We used this platform to examine comprehensive point mutant libraries of both full-length SHP2 (SHP2_FL_) and its isolated phosphatase domain (SHP2_PTP_). These experiments have revealed several mechanistically distinct classes of mutations that provide new insights into the molecular determinants of inter- and intra-domain allostery in SHP2. Our datasets recapitulate the well-known mutational sensitivity at the canonical autoinhibitory interface and show how mutations outside of this interface can indirectly disrupt autoinhibition. Complemented by molecular dynamics simulations, our mutational scans provide clues into the transition between autoinhibited and active states of SHP2 and pinpoint key interactions that likely stabilize the active state(s). Our analyses also delineate the key determinants of catalytic activity and active site conformational fluctuations in the isolated SHP2 phosphatase domain. Notably, this investigation has also produced the first comprehensive map of functional effects for hundreds of previously reported but uncharacterized human SHP2 variants.

## Results

### Rescue of yeast growth from tyrosine kinase toxicity enables SHP2 deep mutational scanning

We developed a yeast viability assay in which cell growth is dependent on the catalytic activity of SHP2. Lacking significant endogenous tyrosine kinase/phosphatase signaling, yeast (*S. cerevisiae*) proliferation is arrested when expressing an active tyrosine kinase, whereas co-expression of an active tyrosine phosphatase can rescue yeast growth^36–38^. We co-expressed SHP2 variants known to have different levels of catalytic activity with two active versions of Src kinase, full-length viral Src (v-Src_FL_) and the isolated c-Src kinase domain (c-Src_KD_). In the presence of either kinase, the rate of yeast growth was dependent on the catalytic activity of the SHP2 variant (Figure S1). Moreover, the activity of the tyrosine kinase dictates the selection pressure of the assay. With the highly active v-Src_FL_ kinase, the more active SHP2 variants were better differentiated, while with the less active c-Src_KD_ kinase, lower activity SHP2 variants were easier to differentiate (Figure S1B,C).

**Figure 2.**
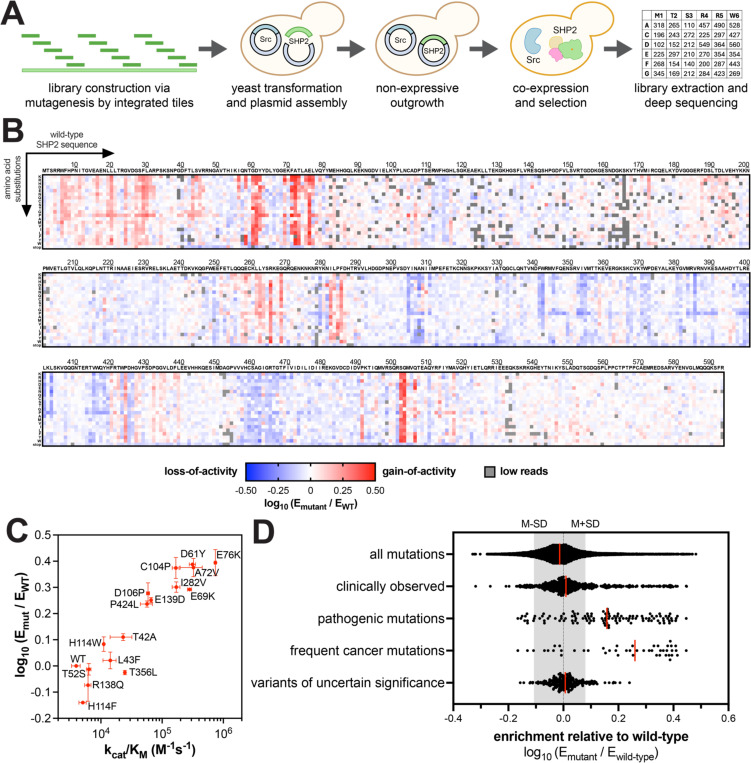
Deep mutational scanning of full-length SHP2. (**A**) Workflow for SHP2 mutational scanning. SHP2 variant libraries were constructed using MITE, integrated into a yeast plasmid, co-expressed with Src, and subject to selection and deep sequencing. (**B**) Heatmap depicting the enrichment scores for SHP2_FL_ co-expressed with c-Src_KD_ (n = 2–4). (**C**) Correlation between SHP2 variant enrichment scores in the SHP2_FL_ + c-Src_KD_ selection assay and measured catalytic activity against DiFMUP (n = 3). (**D**) Enrichment score distributions for different subsets of mutations in the SHP2_FL_ + c-Src_KD_ selection assay. The red line indicates the median of the distribution, and the shaded gray region indicates enrichment within one standard deviation from the median for all mutations in the library. Full SHP2_FL_ mutational scanning datasets, including clinical annotations, can be found in Table S2.

We constructed SHP2 scanning mutagenesis libraries, using Mutagenesis by Integrated TilEs (MITE)^39^, dividing the full-length SHP2-coding gene into 15 separate tiles, each encoding all possible single mutations along different regions of SHP2 (Table S1). Tiles were synthesized as oligo-pools, amplified, and incorporated into the full-length SHP2 gene in a yeast expression vector generating 15 SHP2 saturation mutagenesis sub-libraries covering the entire protein sequence (Figure 2A). These SHP2 sub-libraries were separately introduced into yeast cells alongside plasmids encoding either v-Src_FL_ or c-Src_KD_. Cells were then subject to selection by induction of kinase and phosphatase expression, followed by a 24 hour outgrowth phase. Before and after outgrowth, the SHP2-coding DNA was isolated and subject to deep sequencing, allowing for the calculation of enrichment scores for each variant, relative to wild-type SHP2 (see Methods for calculation). Two to four replicates of high-quality selection data were acquired for each sub-library (each tile), with good correlation of enrichment scores between replicates (Figure S2A). The average enrichment scores of all variants across replicates were plotted as heatmaps (Figure 2B, Figure S2B, and Table S2).

Since wild-type SHP2_FL_ has low basal activity, loss-of-function mutations were not very well represented under high selection pressure in the presence of v-Src_FL_ (Figure S2B). However, at lower selection pressure with c-Src_KD_, both gain- and loss-of-function SHP2 mutants were detected (Figure 2B). This is reflected in the correlation plot of enrichment scores from the two datasets (Figure S2C). While scores were generally well correlated, points in the negative region were more spread out along the c-Src_KD_ axis because of the better detection of depleted variants relative to wild-type SHP2. To validate that the enrichment scores faithfully report on SHP2 phosphatase activity, we purified several full-length SHP2 mutants and measured their catalytic efficiencies (k_cat_/K_M_) against the fluorogenic small molecule substrate DiFMUP. Measured catalytic efficiencies correlated well with the enrichment scores obtained from the deep mutational scans (Figure 2C and Table S3).

Next, we compared our enrichment scores with clinical data of known SHP2 mutants (Figure 2D and Figure S2D). The deep mutational scanning results show a broad range of gain- and loss-of-function across the whole library. Mutations annotated as pathogenic tended to be more gain-of-function, however many pathogenic mutations did not enhance SHP2 catalytic activity. High-frequency cancer mutations skewed further toward gain-of-function, but it is noteworthy that a few of these were not activating, or even loss-of-function, indicating that SHP2 might contribute to pathogenic signaling in ways that are not reflected in our experiment. For example, the S189A mutation, found in some bladder and digestive-tract cancers, is neutral in our datasets, but S189K/R mutations are activating (Figure S2E). Phosphorylation of S189 by Protein Kinase A inhibits SHP2 function^40^, and this could stabilize the autoinhibited state through electrostatic interactions (Figure S2F). Thus, the S189A mutation may operate by subverting inhibitory phosphorylation in some cancer cells, and this is not relevant in our yeast assay. Finally, we found that only a few variants of uncertain significance had a measurable gain- or loss-of-function (Figure 2D and Table S2), suggesting that most uncharacterized variants either are passenger mutations, alter non-enzymatic functions of SHP2, or alter SHP2 activity only in a specific context.

### Mutational scanning maps hotspot residues at the autoinhibitory interface of SHP2

**Figure 3.**
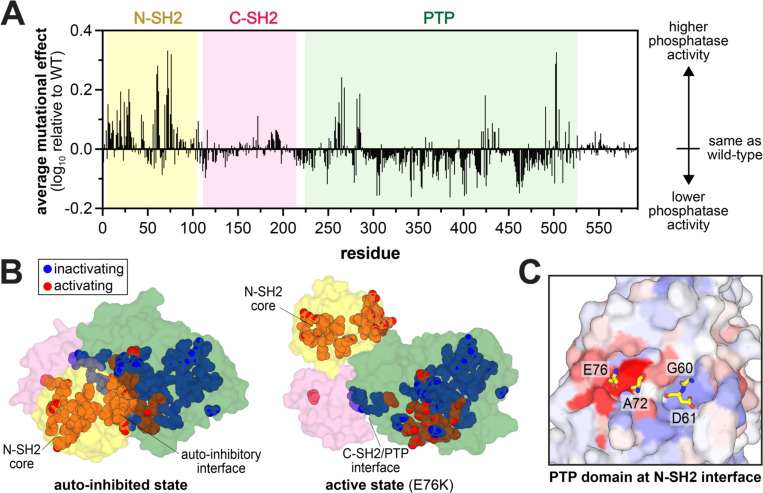
SHP2 mutation distribution. (**A**) Average mutational effects at each residue in SHP2_FL_ in the c-Src_KD_ selection assay. (**B**) Spatial distribution of substantially activating and inactivating SHP2 mutant sites on the autoinhibited and active state structures of SHP2 (PDB codes 4DGP and 6CRF, respectively). Activating mutation sites are colored red, and inactivating mutation sites are colored blue (**C**) Mutational sensitivities in two key PTP domain pockets at the N-SH2/PTP autoinhibitory interface (PDB code 4DGP). Key residues on the N-SH2 domain are shown as yellow sticks. The PTP domain surface is colored based on the average enrichment score from panel A, with red = activating, white = neutral, blue = inactivating.

Next, we calculated the average enrichment scores at each residue on SHP2 sequence (Figure 3A). Most of the sites with highly activating mutations are clustered on the N-SH2 and PTP domains, while sites with highly inactivating mutations are largely confined to the PTP domain. The disordered tail was largely devoid of mutational effects, suggesting that regulation by tail phosphorylation is not operative in our assay^15^. Residues with high mutational sensitivity tended to be highly conserved across SHP2 proteins in metazoans (Figure S3A and Table S4). We then mapped the most mutationally sensitive sites onto the SHP2 autoinhibited and open/active structures (Figure 3B). As expected, many gain-of-function mutations are located on the N-SH2/PTP domain interface, and they likely activate SHP2 by disrupting autoinhibition. Most loss-of-function mutations are buried in the PTP domain and likely impact SHP2 by disrupting catalytic domain stability or active site conformation. Remarkably, the C-SH2 domain was largely devoid of highly sensitive sites, other than a few, discussed in subsequent sections.

Our datasets provide an unbiased assessment of the residues that regulate phosphatase activity, unlike cancer mutation hotspots which are constrained by genetic accessibility and sufficient sampling. In our experiments, the strongest activating mutation sites were at residues G60, D61, A72, and E76 in the N-SH2 domain and R265, S502, and G503 in the PTP domain. These residues cluster into two hotspots at the N-SH2/PTP interface when the protein is in the autoinhibited conformation. The first hotspot is defined by a pocket in the PTP domain, lined by R265, S502, and G503, which buries A72 and E76 from the N-SH2 domain (Figure 3C and Figure S3B). As expected, given that these are known cancer mutation sites, mutations at these residues display significant basal activity increases in biochemical assays (Figure 2C). The other hotspot consists of G60 and D61 on an N-SH2 loop reaching into a pocket on the PTP domain made up of several positively charged residues, including R362, K366 and R465 (Figure 3C and Figure S3C). Unlike the PTP domain pocket that binds to A72 and E76, which has activating mutations, the G60 and D61 binding pocket seem to be more inactivating upon mutation, likely because those PTP domain residues are also key to catalysis.

### Destabilization of the N-SH2 domain activates SHP2

**Figure 4.**
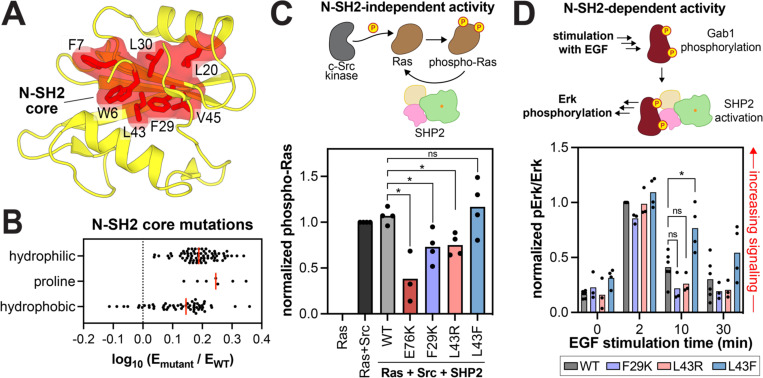
Effects of mutations in the N-SH2 domain core. (**A**) Hydrophobic core residues in the N-SH2 domain (PDB code 1AYB). (**B**) Distribution of mutational effects at the residues shown in panel A. Hydrophilic amino acids include C, D, E, H, K, N, Q, R, S, and T; hydrophobic amino acids include A, F, G, I, L, M, V, W, Y. The red line indicates the median enrichment for all mutations in each group. (**C**) N-SH2-independent catalytic activity of SHP2 N-SH2 core mutants and E76K in a Ras dephosphorylation assay in HEK293 cells (n = 3). Phospho-Ras levels are normalized to total Ras levels and to phospho-Ras in the Ras+Src sample. (**D**) Downstream signaling activity of SHP2 N-SH2 core mutants, dependent on N-SH2 binding to Gab1, in HEK293 cells stimulated with epidermal growth factor (EGF). Phospho-Erk levels are shown, normalized to total Erk levels and to the 2 minute time point for the wild-type SHP2 sample (n = 3–4). In panels (C) and (D), * denotes P < 0.05.

Our mutational scans revealed one large cluster of activating mutations outside of the autoinhibitory interface (Figure 3B). In the N-SH2 domain, the residues that make up the hydrophobic core (W6, F7, I11, A16, L20, F29, L30, L43 and V45) are enriched in activating mutations (Figure 4A,B). We hypothesize that mutations at these sites disrupt hydrophobic packing, thereby destabilizing the N-SH2 domain and weakening its autoinhibitory binding to the PTP domain. Consistent with this hypothesis, polar mutations at these sites are more activating than nonpolar mutations, as are proline substitutions which likely destabilize the central N-SH2 β-sheet. (Figure 4B). Notably, the clinically observed L43F mutation mildly enhances basal SHP2 catalytic activity and shows lower melting temperature for the full-length protein by differential scanning fluorimetry, indicating weakened autoinhibition (Figure S4A)^26,41^. The isolated N-SH2 domain with the L43F mutation also has lowered stability, demonstrated by its lowered melting temperature (Figure S4B), but is still competent to bind phosphopeptides^26^.

Although non-conservative N-SH2 core mutations can dramatically activate SHP2, they appear to be rare in human diseases. The aforementioned L43F mutation is one of the few documented pathogenic mutations in the N-SH2 core. Less conservative mutations such as L43P and L43R, which are substantially more activating, are not observed in ClinVar, despite being accessible by a single nucleotide substitution in the human SHP2-coding gene (*PTPN11*) (Figure S4C)^24^. While hyper-destabilizing N-SH2 core mutations can activate SHP2 by relieving autoinhibition, they most likely also disrupt the phosphoprotein binding functions of the N-SH2 domain. Thus, such mutants would not be able to engage in human signaling pathways that require N-SH2 binding capabilities. Critically, these pathways are not operative in our yeast selection assay, and the phosphoprotein binding function of the N-SH2 domain is probably expendable in this context.

To test the hypothesis that N-SH2 core mutations activate SHP2 but disrupt N-SH2 binding functions, we initially attempted to purify from *E. coli* full-length SHP2 or the isolated N-SH2 domain containing non-conservative core mutations. We were unable to produce these proteins, likely because these mutations are severely destabilizing. Instead, we expressed and assayed the hyperactive F29K and L43R mutants alongside the mildly-activating L43F mutant in two cellular assays for SHP2 activity, one that depends on N-SH2 function and one that does not. First, we examined the ability of the SHP2 mutants to dephosphorylate N-Ras that has been phosphorylated by co-expressed Src kinase^42^. This activity is not thought to depend on SH2 interactions (Figure 4C). Both F29K and L43R dephosphorylated N-Ras more efficiently than wild-type SHP2, whereas L43F was comparable to wild-type SHP2, consistent with the mutational scanning data (Figure 4C and Figure S4D). Next, we tested the signaling capabilities of these mutants in a context where N-SH2 binding functions are required. SHP2 was co-expressed with Gab1, a known binding partner, cells were stimulated with the epidermal growth factor, and Erk phosphorylation was monitored as a downstream marker of SHP2-mediated activation of this pathway (Figure 4D)^26^. Canonical hyperactivating mutations in SHP2, like E76K, dramatically enhance Erk phosphorylation in this assay^26^. By contrast, the F29K and L43R mutants did not enhance Erk phosphorylation over wild-type SHP2, despite their increased catalytic activities (Figure 4D and Figure S4E). These results suggest that, although destabilization of the N-SH2 core can activate SHP2, excessive destabilization can disrupt N-SH2 function and precludes downstream signaling. This likely explains why non-conservative N-SH2 core mutants are not frequently found in human diseases, despite their high catalytic activity.

### C-SH2/PTP interactions facilitate release from autoinhibition and stabilize activated SHP2

**Figure 5.**
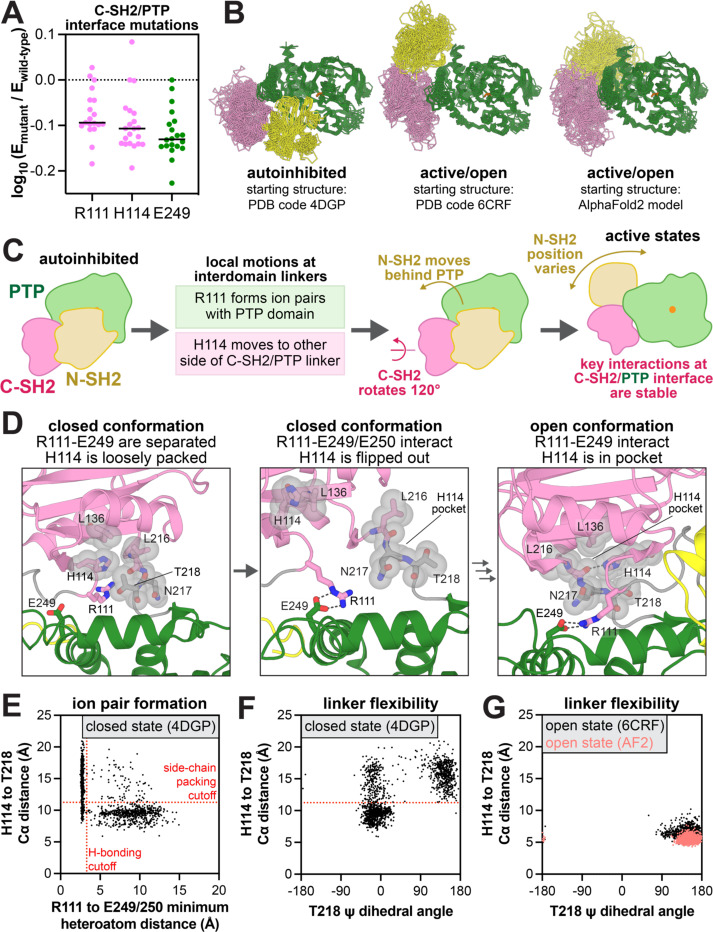
Structure and dynamics at the C-SH2/PTP interface. (**A**) Mutational effect at R111, H114, and E249 in the SHP2_FL_ selection with c-Src_KD_. (**B**) Conformational sampling across 18 MD simulations of SHP2 starting from three different conformational states. (**C**) Hypothesized sequence of events in the SHP2 closed-to-open transition. (**D**) Representative frames from MD simulations of SHP2 highlighting a lack of R111-E249 ion pairing and loose packing of H114 in simulations starting from the closed conformation (*left*), formation of the R111-E249 ion pair with concomitant exit of H114 from the C-SH2/PTP linker pocket in simulations starting from the closed conformation (*middle*), and stable R111-E249 and H114-L216 interactions in simulations starting from the open conformation (*right*). The transition from the middle to right frame is not observed in our simulations. (**E**) Correlation between R111-E249/E250 distance (shortest distance between one of the arginine terminal nitrogens and one of the four glutamate carbonyl oxygens) and H114-T218 Cα distance in the SHP2 closed conformation simulations. (**F**) Correlation between T218 ψ dihedral angle and H114-T218 Cα distance and in the SHP2 closed conformation simulations. (**G**) Correlation between R111-E249/E250 distance and H114-T218 Cα distance in the SHP2 open conformation simulations.

Very few positions in the C-SH2 domain have strong activating mutations. However, we identified a cluster of residues near the C-SH2/PTP interface, namely R111 and H114 in the C-SH2 domain and E249 in the PTP domain, where most mutations are significantly inactivating (Figure 5A). In the open state crystal structure of SHP2 E76K, these residues participate in intimate interactions – R111 forms a stable ion pair with E249 on the PTP domain, and H114 docks in a pocket consisting of L216, N217, T218 on the C-SH2/PTP linker, as well as L136 (Figure S5A). These interactions are disassembled in crystal structures of the autoinhibited state – R111 points away from the PTP domain, and H114 interacts loosely with the side chain of T218 instead of docking in a pocket made up of the surrounding residues (Figure S5B). These structural differences, coupled with the strongly deactivating mutations at R111, H114, and E249, suggest that these residues play a key role in SHP2 activation. Notably, the R111-E249 ion pair was thoroughly characterized in a recent study, which demonstrated that this interaction stabilizes the active state of SHP2^43^.

To better understand the role of H114 and the R111-E249 ion pair in SHP2 activation, we performed molecular dynamics (MD) simulations on near-full-length SHP2 (excluding the disordered tail), starting from three different conformational states: the autoinhibited conformation (PDB code 4DGP), the open state observed in a crystal structure of the E76K mutant (PDB code 6CRF), and an alternative open state observed in an AlphaFold2 model of SHP2, in which the N-SH2 domain is further behind the PTP domain when compared to the crystal structure (Figure 5B)^44^. We opted to use these two distinct open conformations given the evidence suggesting that SHP2 adopts multiple active conformational states^18–20^. Starting from each state, we conducted three 2.5 μs simulations with both the wild-type sequence and the E76K mutant. Although our simulations were too short to sample a full transition between the autoinhibited and open states, we observed local motions at the inter-domain interfaces that provided insights into how this transition may be initiated (Figure 5C,D).

In the open conformation trajectories starting from both the crystal structure and AlphaFold2 model, H114 is docked stably in the aforementioned pocket made by L216, N217, T218, and L136. This packing is stabilized by a hydrogen bond between the H114 side chain and L216 backbone (Figure S5C). Notably, the H114W mutant, which can also make a hydrogen bond, enhanced activity in the mutational scans and in an in vitro phosphatase assay, whereas H114F reduced *k*_cat_, suggesting that both hydrogen bonding capability and hydrophobicity are important at this position (Figure S5D and Table S3). Additionally, in all of the open conformation simulations, R111 frequently forms ion pairs with E249 or the adjacent E250, with occasional excursions to interact with the proximal residue E232 (Figure S5E,F). The persistence of these R111 and H114 interactions in both sets of open conformation simulations is consistent with our mutational scanning data and supports the notion that both the crystal structure and AlphaFold2 structure are plausible models for active SHP2.

The C-SH2/PTP interface is more dynamic in the closed conformation simulations, and motions in this region suggest a plausible sequence of events for the initiation of SHP2 activation (Figure 5C). In the starting structures, the side chains of H114 and T218 are packed against one another, stabilizing the C-SH2/PTP linker where T218 resides, while R111 is pointed away from the PTP domain (Figure 5D, *left panel*). During the simulations, R111 rotates into the C-SH2/PTP cleft and intermittently forms ion pairs with E249 or E250, despite the lack of large inter-domain rearrangements (Figure 5D, *middle panel*). When R111 interacts with E249/E250, this movement disrupts the interaction between H114 and T218 (Figure 5D, *middle panel*, Figure 5E). This, in turn, increases C-SH2/PTP linker dynamics, as evidenced by the ability of T218 and other linker residues to adopt multiple backbone conformations (Figure 5F). This is in stark contrast to the open conformation trajectories, where H114 intimately binds to L216, N217, and T218, and these linker residues are more rigid (Figure 5D, *right panel*, Figure 5G).

We hypothesize that the increase in C-SH2/PTP linker flexibility and the release of H114 are essential early steps for SHP2 activation – the C-SH2 domain must rotate relative to the PTP domain to transit from the autoinhibited to active state (Figure 5C), and H114 must dissociate from one side of the linker and dock stably on the other side (Figure 5D). Mutational effects at T218 support this mechanism: bulky and hydrophobic mutations, which should strengthen hydrophobic interactions with H114, are inactivating, whereas small residue substitutions, including clinically observed variants of uncertain significance T218S and T218A, which likely increase linker flexibility, are activating (Figure S5G).

### Isolated linker and C-SH2 mutations activate SHP2

There are a few residues in the SH2 domain regions where we observed unique mutational signatures, but a mechanistic explanation remains elusive. For example, in the linker connecting the N-SH2 and C-SH2 domains, mutations of C104 and D106 to proline are activating, both in the selection assays and in biochemical measurements (Figure 2C, Figure S6A,B, and Table S3). These effects strongly suggest that there are conformational constraints on this linker in the SHP2 closed conformation. However, unlike many other activating mutations, these mutations do not cause a dramatic reduction in melting temperature via differential scanning calorimetry (Figure S6C)^41^. Thus, how the N-SH2/C-SH2 linker regulates SHP2 activity remains unclear. Another pair of anomalous residues are R138 and E139 in the C-SH2 domain. R138Q and E139D are known to be pathogenic^45,46^. In our mutational scanning data, E139D is activating in both SHP2_FL_ selection assays (with v-Src_FL_ and c-Src_KD_), and this mutation is known to activate SHP2 in biochemical assays as well. Remarkably, only E139D, but no other substitutions at this position, activate SHP2 (Figure S6D). Similarly, R138E/Q are the only activating mutations at this position, but in this case, they only appear to be activating in the v-Src_FL_ screen (Figure S6E). We previously showed that R138Q severely diminishes phosphopeptide binding to the C-SH2 domain but has no impact on basal catalytic activity^26^. Differential scanning fluorimetry shows that both E139D and R138Q slightly destabilize full-length SHP2, consistent with activation,^26^ but only R138Q substantially destabilizes the isolated C-SH2 (Figure S6C). Notably, very few hydrophobic core mutations in the C-SH2 domain activate SHP2, in stark contrast to the N-SH2 domain. Thus, the precise role of C-SH2 stability on SHP2 regulation requires further investigation.

### The tyrosine phosphatase domain of SHP2 has distinct mutational sensitivity in the absence of regulatory domains

**Figure 6.**
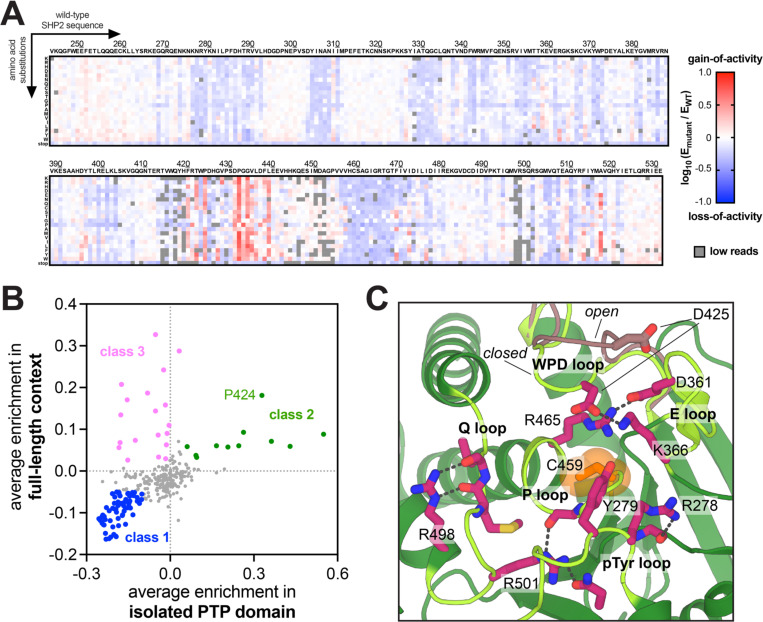
Mutational scanning of isolated SHP2 PTP domain reveals key buried charged residues for catalysis (**A**) Mutational scanning heatmap of SHP2 PTP domain selected in the v-Src FL screen. Full SHP2_PTP_ mutational scanning datasets can be found in Table S5. (**B**) Correlation between SHP2 FL, c-Src KD selection and SHP2 PTP, v-Src FL selection. PTP domain residues are classified into three classes: Class 1: residues with consistently inactivating effects in both screens; Class 2: residues with consistently activating effects in both screens. Class 3: residues with opposite mutational effects. (**C**) Network of electrostatic and hydrogen bond interactions between buried, charged and polar amino acids in the PTP domain that stabilize loops in the active site (PDB code 6CMQ, chain D). The pTyr loop, E loop, WPD loop, P loop, and Q loop are colored in light green. The WPD loop open conformation is colored brown (PDB code 6CMQ chain C). Key residues are colored in purple.

The catalytic activity of the PTP domain in SHP2 is regulated both by SH2 domain-mediated autoinhibition and by intra-domain interactions. To dissect the contributions of inter- and intra-domain regulation, we also conducted mutational scanning on SHP2_PTP_ and compared these results with the SHP2_FL_ datasets. Because the isolated PTP domain has high basal activity, we do not detect gain-of-function mutations under the lower selection pressure in the presence of c-Src_KD_ (Figure S7A and Table S5). With v-Src_FL_, which provides a higher selection pressure, both enriched and depleted mutations are clearly detected (Figure 6A and Table S5). For both datasets, mutational sensitivity correlates well with sequence conservation across the human PTP domains (Figure S7B and Table S4). To compare the mutational sensitivities of the PTP domain in both full-length and truncated SHP2 screens, we correlated the position-averaged enrichment scores for the SHP2_FL_/c-Src_KD_ selection with the SHP2_PTP_/v-Src_FL_ selection. We observed three main classes of mutation sites: (1) sites with inactivating mutations that are depleted in both screens; (2) sites with activating mutations that are enriched in both screens; (3) sites with mutations that inactivate the PTP domain but activate full-length SHP2, likely by disrupting autoinhibition (Figure 6B). Each class reveals key factors of SHP2 intra- and inter-domain regulation.

### Buried electrostatic interactions are critical for the structural integrity of the SHP2 catalytic site

Mutations that are depleted in both the SHP2_FL_ and the SHP2_PTP_ selection assays reveal key determinants of its catalytic activity (Figure 6B,C). For example, we observed many loss-of-function mutations at well-known functionally important residues, such as C459, the catalytic cysteine, R465, which is critical for coordinating the phosphoryl group on the substrate, and Y279, which provides a key binding interface for the substrate phosphotyrosine. Less-studied substrate binding residues, including N281 and H426, were also found to be sensitive to mutations. Their corresponding residues on PTP1B, D48 and F182, have been shown to engage a backbone carbonyl and the phosphotyrosine phenyl ring on phosphopeptide substrates^47^, indicating N281 and H426 play a role in substrate recognition in SHP2 as well (Figure S7C). Notably, many H426 mutations have been clinically observed, including in cancer, indicative of the physiological importance of this residue.

Surrounding the substrate binding site, key loops including the phosphotyrosine recognition loop (pTyr loop), E loop, WPD loop, catalytic loop (P loop), and Q loop construct the catalytic site. These loops sit on top of the PTP domain central β sheet, wrapped around a central helix α2. As the structural scaffold of the PTP domain, interactions between the β sheet and helix α2 are critical, and mutations in this region are not tolerated (Figure 6A and Figure S7D). On the other side of the β sheet, several buried charged residues (R278, E361, K366, R465, R498 and R501) appear consistently inactivating upon mutation in both the SHP2_FL_ and SHP2_PTP_ selection assays (Figure 6C and Figure S7E). The invariability of these charged residues indicates their structural importance in organizing the catalytic center. Some, but not all, of these key interactions are corroborated by previous studies: the E loop E361 interacts with R465 on the catalytic loop, positioning it at a favorable conformation for substrate recognition^48^. On the same loop, K366 mediates the active WPD loop closed state by forming an interaction with the catalytic D425 (Figure 6C and Figure S7F)^49,50^. We also identified some lesser-known interactions that are also crucial for SHP2 activity. On the pTyr loop, R278 dictates loop positioning by hydrogen bonding with backbone carbonyl of G332, stabilizing the substrate binding Y279 in the catalytic center (Figure 6C and Figure S7G). The buried R501 on the Q loop holds both the P and Q loop down to the central β sheet by interacting with N306 and the backbone of G462, while R498 hydrogen bonds with M504 and V505 backbone, collectively constraining the conformations of these loops for catalysis (Figure 6C and Figure S7H). Together, all these interactions form an electrostatic and hydrogen bonding network that brings all the catalytic loops together to an enzymatically active conformation.

### Active site residue identities are constrained by a trade-off between regulation and catalysis

**Figure 7.**
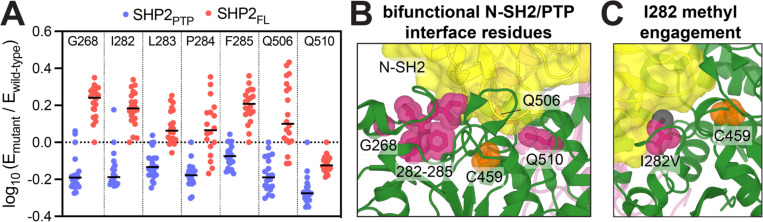
PTP domain mutational effects that disrupt both autoinhibition and catalysis. (**A**) Enrichment score distributions for several residues where mutations inactivate SHP2_PTP_ by disrupting catalysis (blue dots) and activate SHP2_FL_ by disrupting autoinhibition (red dots). Q506 is inactivating in both contexts and shown for comparison. (**B**) Rendering of N-SH2/PTP interface, highlighting G268, I282-F285, Q506, and Q510 (shown in red spheres). G268 and I282-F285 control pTyr loop conformation, whereas Q506 and Q510 are directly involved in catalysis. Q506 is located behind the N-SH2 domain, which is colored yellow (PDB code 4DGP). (**C**) The I282V mutation does not disrupt pTyr loop conformation, but the extra methyl group on isoleucine relative to valine (black sphere) is crucial for N-SH2 binding in the autoinhibited state.

Many catalytic residues in the PTP domain of SHP2 also lie at the N-SH2/PTP autoinhibitory interface. For example, the D61 binding pocket, discussed above (Figure 3C), consists of positively charged residues K366 and R465 on the PTP domain, which are also key residues on catalytic loops (Figure 6C). Because of their importance for phosphatase activity, mutations at these residues are not activating in the SHP2_FL_ screen, although they likely disrupt the autoinhibition. Residues around the E76 pocket can also contribute both to catalytic activity and autoinhibition. Mutations at G268 and residues I282-F285 actually have opposite mutational effects in the SHP2_FL_ and SHP2_PTP_ selection assays (Figure 6B, *class 3*, and Figure 7A). These residues are located close to the pTyr loop, and mutations at these sites likely disrupt substrate binding, thereby decreasing phosphatase activity. In the full-length construct, however, the same mutations disrupt E76 docking onto the PTP domain (Figure 7B). The resulting destabilization of the autoinhibited state compensates for partial loss of activity in the PTP domain, yielding net activating effects in SHP2_FL_, contrary to the inactivating effects in SHP2_PTP_ (Figure 7A). Interestingly, among the I282 mutations, a known pathogenic mutation, I282V, is activating in both constructs, whereas all other I282 substitutions activate SHP2_FL_ but inactivate SHP2_PTP_ (Figure S8A). The extra methyl group on isoleucine relative to valine is important for the hydrophobic interaction with the N-SH2 domain, explaining why even the conservative I282V mutation activates SHP2_FL_ (Figure 7C). By contrast, only I282V, and no other I282 mutations, can fully preserve the catalytically competent conformation of the pTyr loop.

The Q loop, which presents key catalytic glutamine residues, is also located on the autoinhibitory interface while facilitating catalysis. Specifically, Q510 and Q506 are thought to be the key catalytic residues in the formation and hydrolysis of the phospho-enzyme intermediate^51^. Surprisingly, despite their reported crucial roles in catalysis, only Q510 displays invariably strong loss-of-function mutational effects (Figure 7A). Q506 mutations, by contrast, appear to be activating in the SHP2_FL_ screens, suggesting that this residue both contributes to autoinhibition and is expendable for catalysis. Furthermore, in the SHP2_PTP_ screens, while mutations at both sites are generally detrimental, Q510 mutations have stronger loss-of-function effects (Figure 7A and Figure S8B,C). This suggests that Q510 is indispensable for catalysis, as it is likely required both for formation and hydrolysis of the phosphor-enzyme intermediate. On the other hand, Q506 mutations may only affect the hydrolysis step, with an overall marginal impact on PTP activity^51^. In the SHP2_FL_ construct, the decrease of activity upon Q506 mutations can be compensated by disruption of autoinhibition, while the net mutational effects of Q510 are still inactivating due to its essentiality for catalysis.

### Residues with activating mutations in SHP2_PTP_ govern WPD loop motions

**Figure 8.**
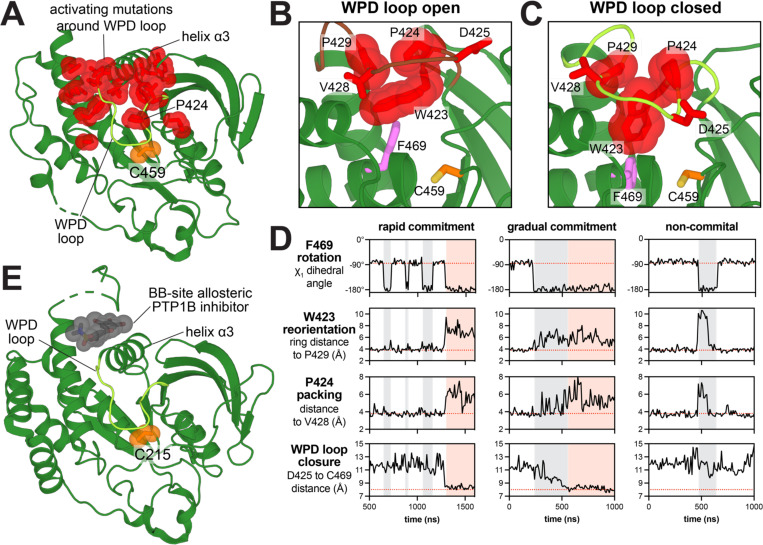
Mutational effects that alter WPD loop conformational dynamics. (**A**) Residues with consistent gain-of-function mutational effects around the WPD loop (PDB code 3ZM0). (**B**) Frame from MD trajectory of SHP2 starting from PDB 6CRF, showing packing of W423, P424, P429, and F469 in WPD loop open conformation. F469 points towards the WPD loop in this structure. (**C**) Frame from an MD trajectory starting from PDB 6CRF, showing that, in the WPD closed conformation, W423 displaces F469, and P424 is released from its intimate packing in the turn of the loop. (**D**) Three MD trajectories starting from the open conformation, showing coordinated movements between F469, W423, and P424. The first trajectory is an example of these movements culminating in WPD closure. The second trajectory also shows WPD loop closure, but more gradually. The third trajectory shows coupled F469, W423, and P424 motions that revert back to their starting state, resulting in no WPD loop closure. F469 rotation is quantified by χ_1_ angle of this residue. W423 reorientation is quantified by the distance between W423 Cζ_3_ and P429 Cγ. P424 packing is quantified by the distance between P424 Cγ and the V428 carbonyl. WPD loop closure is quantified by the distance between the D425 and C459 Cα atoms. Gray shaded segments denote F469 in a permissive state for WPD loop closure. Pink shaded segments denote WPD loop closure. (**E**) Structure of PTP1B bound to BB site allosteric inhibitor (PDB code 1T48).

Consistently activating mutations in both SHP2_FL_ and SHP2_PTP_ selection assays cluster around the WPD loop (Figure 6B, *class 2*, and Figure 8A). These mutations likely enhance activity by inducing WPD loop closure or altering WPD loop dynamics, both of which are crucial for SHP2 catalysis.^52,53^ Notably, the closed, catalytically-competent WPD loop conformation in tyrosine phosphatases is incompatible with the SHP2 autoinhibited conformation, as the loop would clash with the N-SH2 domain (Figure S9A). In WPD loop closed state, the catalytic D425 occupies the same pocket that is otherwise occupied by D61 in the autoinhibited state, sitting between R362, K366 and R465 (Figure S9B). Thus, mutations that favor WPD loop closure not only enhance catalytic activity by promoting catalysis, but they also drive SHP2 opening, which is why they are enriched in both SHP2_FL_ and SHP2_PTP_ screens.

In the aforementioned MD trajectories of the active conformations of SHP2, we captured several instances of the WPD open-to-closed transition. These events reveal coordinated movements between key residues in this region and provide insights into how SHP2 may differ from other phosphatases, such as PTP1B (Figure 8B-D). When SHP2 adopts the WPD open conformation, W423, P424, and P429 pack together via hydrophobic interactions, and this cluster is buttressed by F469 (Figure 8B). Notably, F469 adopts multiple rotameric states in crystal structures and in our MD trajectories (Figure 8D and Figure S9C)^54^. In the transition to the WPD closed conformation, F469 rotates away from the WPD loop, and W423 moves away from the loop center into the cleft that is otherwise occupied by F469 (Figure 8C). This transition releases P424 from its packing interactions, allowing it to access more diverse conformations. We quantified these movements by measuring the distance between W423 Cζ_3_ and P429 Cγ, the χ_1_ angle of F469, and the distance between P424 Cγ and the V428 carbonyl. Our analysis shows that the F469 rotation acts as a gatekeeper for W423 movement and WPD loop closure (Figure 8D). In our simulations, we observed instances of concerted W423/F469/P424 movements that ultimately culminated in WPD loop closure, as well as instances where these residues sampled an on-pathway state and then reverted back to their inactive conformation, thereby aborting WPD loop closure (Figure 8D). A key feature of WPD loop rearrangement in the simulations is that the side chain positioning of P424 becomes less constrained. Notably, every mutation at P424 was activating in deep mutational scans with SHP2_PTP_ and SHP2_FL_, and the P424L mutation results in Noonan Syndrome (Figure 2B and Figure 6A,B)^24^. Basal activity and melting curve measurements confirm that P424L activates SHP2 and destabilizes its autoinhibited state (Figure 2C, Figure S9D, and Table S3), supporting the idea that disruption of P424 packing allows the WPD loop to close more easily. On the other hand, W423 and P429 mutations were inactivating in our experiments, likely due to the loss of stabilizing interactions in the WPD closed state (Figure 6A).

Similar WPD loop dynamics have also been observed for PTP1B and are most likely conserved across tyrosine phosphatases^52^. However, our MD simulations and mutagenesis point to key differences between PTP1B and SHP2. For example, in both WPD open and closed structures, F225 in PTP1B, which corresponds to F469 on SHP2, always points away from the WPD loop, in the orientation that is consistent with WPD loop closure in SHP2^52^. The locked rotameric state for F225 in PTP1B is due to steric hindrance by L110, which prohibits F225 rotation and may predispose PTP1B toward an active state (Figure S9C). Indeed, the residue in SHP2 corresponding to PTP1B L110 is a threonine (T356), and bulky aliphatic mutations (I, L, V) at T356 increase SHP2 activity, supporting the idea that restrained F469 movement favors WPD closure (Figure S9E and Table S3). Furthermore, F225 has been reported to play central role in PTP1B stability, with conservative mutations (Y, I, L) destabilizing the enzyme, and alanine at this position yielding insoluble protein^55^. By contrast, several large and small substitutions at this site in SHP2, including F469Y and F469A, are activating (Figure S9F). These subtle differences in packing and dynamics around the WPD loop are likely to be key determinants underlying differences in phosphatase activity and regulation across PTPs.

Our mutational scans also reveal commonalities between SHP2 and PTP1B. On the back of the WPD loop, a group of residues show strong activating effects in the SHP2_PTP_ selection and are also modestly activating in the SHP2_FL_ context (Figure 8A). These residues, which include D395, Y396, F420, T422 and D431-L439, most likely dictate the positioning of helix α3. Allosteric inhibitors of PTP1B have been identified that bind to a pocket in this region, termed the ‘BB site’ (Figure 8E)^21,56^. Those inhibitors operate by inducing rotation in helix α3 that propagates to the contiguous WPD loop, and it has been shown that the ‘BB site’ inhibitor BBR can also inhibit SHP2. ^52,57^ Our results provide additional evidence that the allosteric network connecting the BB site to the active site is conserved in SHP2 and support the idea that SHP2 activity could be modulated through ligand binding in this region.

## Discussion

In this study, we used a yeast selection assay to compare the catalytic activities of nearly all possible point mutants in the human tyrosine phosphatase SHP2. We conducted deep mutational scans of both full-length SHP2 (593 residues, ~12,000 variants) and its isolated catalytic domain (289 residues, ~6000 variants). Thus far, our understanding of SHP2 regulation has largely been driven by the characterization of 10–20 disease-associated mutations, most of which cluster at the N-SH2/PTP interface^27,28,58^. The high-throughput mutagenesis datasets presented here reveal several mechanistically distinct classes of mutations that provide new insights into SHP2 inter-domain regulation and also reveal previously unknown determinants of catalytic activity within the phosphatase domain. For example, our results showed that mutations in the core of the N-SH2 domain can activate SHP2 by destabilizing that domain, thereby indirectly disrupting autoinhibition. Given that many multi-domain proteins are regulated by inter-domain autoinhibition^11^, destabilizing mutations in non-catalytic domains may be a common mechanism for dysregulation. Our scanning mutagenesis experiments also identified a cluster of residues at the C-SH2/PTP interface that stabilize the active state of SHP2 and likely play a role in initiating the transition from the autoinhibited to active state. Finally, our results point to mechanisms of intra-domain allostery within the SHP2 catalytic domain that have been observed for other members of the classical tyrosine phosphatase family^21,52^.

Multi-domain proteins are regulated by a combination of inter- and intra-domain interactions. A novel feature of our study, relative to many other deep mutational scanning studies, is that we examined mutational sensitivity in SHP2 both in the presence and absence of regulatory domains. This juxtaposition revealed a trade-off between catalytic activity and autoinhibition. Since SHP2 autoinhibition operates primarily by a regulatory domain (the N-SH2 domain) sterically blocking the active site^13^, mutations on the phosphatase domain that disrupt autoinhibition may also inactivate the enzyme. This functional trade-off likely explains why the strongest activating mutations in SHP2 are found on the N-SH2 domain side of the N-SH2/PTP interface. Intriguingly, mutations in the phosphatase domain that intrinsically enhance catalytic activity by forcing it into a catalytically-competent conformation can also disrupt autoinhibition. Given the extensive potential for allostery within PTP domains^52^, it is likely that poorly characterized pathogenic mutations in the SHP2 PTP that are not at the N-SH2/PTP interface operate via this mechanism (e.g. P424L, discussed earlier, which drives the WPD loop into an active conformation).

The presence of two SH2 domains in SHP2 provides an interesting example of functional specialization within a single signaling protein. All SH2 domains have high structural homology, and the SHP2 N- and C-SH2 domains are more similar to one another than most other SH2 domains^59^. Despite this homology, the N-SH2 and C-SH2 domains in SHP2 participate in inter-domain regulation differently. While the N-SH2 domain governs in SHP2 autoinhibition through extensive interactions with the PTP domain, our results show that only residues on each end of the C-SH2 domain appear to contribute substantially to the SHP2 activation process. Furthermore, N-SH2 stability is a key determinant of autoinhibition, whereas C-SH2 domain stability appears less crucial, as evidenced by the nominal effects of C-SH2 core mutations (Figure 3A,B). These points are relevant to SHP2 activation in the absence of phosphoprotein activators. Both SH2 domains participate in activator binding through their phosphotyrosine recognition capabilities, but again, the responsiveness of SHP2 to activators appears to be more driven by N-SH2 binding than C-SH2 binding^19,26,60^. Nonetheless, the C-SH2 domain contributes to localization^60^, binding specificity^61^, and tuning the activity of SHP2^19,60^. Of note, there are a few pathogenic mutations in the C-SH2 domain, including R138Q and E139D, discussed above. These observations highlight another layer of complexity that has evolved in modular signaling proteins.

SHP2 is the target of several allosteric inhibitors that are in development or currently in clinical trials for the treatment of cancer^62^. Although our deep mutational scanning experiments were not conducted in the presence of SHP2 inhibitors, our datasets can still provide insights into resistance mutations. Established allosteric SHP2 inhibitors all bind to one of two possible sites, and in both cases stabilize the autoinhibited state^62,63^. The ‘tunnel’ binding site lies between the C-SH2 and PTP domains, and the ‘latch’ binding site is at the N-SH2/PTP interface (Figure S10A)^63^. Residues at these two sites show distinct mutational effects in our datasets. The ‘tunnel’ site residues are important for SHP2 activation, and mutations in this region are mostly inactivating or neutral (Figure S10B). The ‘latch’ site residues facilitate autoinhibition and many are activating upon substitution (Figure S10C). Thus, we hypothesize that drugs which bind to the ‘tunnel’ site at the C-SH2/PTP interface will be less susceptible to drug resistant mutations, because mutations that disrupt binding at that site will also inactivate SHP2. By contrast, inhibitors that bind to the ‘latch’ site at the N-SH2/PTP interface will be more susceptible to resistance mutations, as mutations at this site that disrupt drug binding will also activate SHP2. Mutations which broadly destabilize the SHP2 autoinhibited state are likely to cause resistance to drugs that bind either site^18^. Notably, our deep mutational scans revealed an allosteric site on the tyrosine phosphatase domain of SHP2, and this site is already the target of an allosteric inhibitor of PTP1B (Figure 8A,E)^21,56^. These results suggest that intra-domain allostery may be exploitable for SHP2 inhibition, whereas most successful efforts to date have focused on taking advantage of SHP2 inter-domain allostery for pharmacological inhibition^62^.

Several of the mechanistic observations in this study were initially inspired by our large-scale mutagenesis datasets, but they ultimately refined with the support of molecular dynamics simulations. In particular, scanning mutagenesis revealed a cluster of residues at the C-SH2/PTP interface that are critical for SHP2 activity. While these residues are likely to stabilize the active state(s) of SHP2, molecular dynamics simulations and subsequent re-evaluation of mutational sensitivity in this region strongly support the idea that these residues play a role in initiating the transition from the autoinhibited to active state. Similarly, deep mutagenesis revealed an extensive network of residues emanating from the WPD loop to control catalysis, and molecular dynamics simulations clarified how motions in this region propagate to the active site. Thus, our study highlights how mutational scanning experiments can provide insights into protein dynamics. Finally, we note that the yeast selection assay described here can be adapted to investigate mutational effects in other protein tyrosine phosphatases^37,38^. We envision that this platform, particularly when coupled with MD simulations, experimental biochemistry/biophysics, and cell signaling studies, will become invaluable tool to map sequence-structure-function relationships across this important enzyme family.

## Supplementary Material

Supplement 1 **Figure S1.** Yeast growth and selection by co-expression of Src kinase and SHP2 variants.
**Figure S2.** Scanning mutagenesis and selection assays with SHP2_FL_.
**Figure S3.** Hotspot residues at the N-SH2/PTP interface.
**Figure S4.** Destabilization of the N-SH2 domain by core mutations.
**Figure S5.** Mutational sensitivity and dynamics at the C-SH2/PTP interface.
**Figure S6.** Unique mutational effects at linker and C-SH2 residues.
**Figure S7.** Scanning mutagenesis and selection assays with SHP2_PTP_.
**Figure S8.** Mutational effects at I282, Q506, and Q510.
**Figure S9.** WPD loop motions and conformational constraints in SHP2.
**Figure S10.** Mutational sensitivity at allosteric inhibitor binding sites in SHP2.

Supplement 2 **Table S1.** Oligonucleotide pools used for construction of the SHP2 scanning mutagenesis library via MITE.

Supplement 3 **Table S2.** Enrichment scores for SHP2_FL_ election assays with v-Src_FL_ and c-Src_KD_.

Supplement 4 **Table S3.** Catalytic parameters for DiFMUP dephosphorylation by select SHP2 mutants.

Supplement 5 **Table S4.** Sequence conservation scores for metazoan SHP2 proteins and human classical PTP domains.

Supplement 6 **Table S5.** Enrichment scores for SHP2_PTP_ selection assays with v-Src_FL_ and c-Src_KD_.
